# Effects of Pioglitazone on Asymmetric Dimethylarginine and Components of the Metabolic Syndrome in Nondiabetic Patients (EPICAMP Study): A Double-Blind, Randomized Clinical Trial

**DOI:** 10.1155/2013/358074

**Published:** 2013-04-18

**Authors:** Pedram Shokouh, Adel Joharimoghadam, Hamidreza Roohafza, Masoumeh Sadeghi, Allahyar Golabchi, Maryam Boshtam, Nizal Sarrafzadegan

**Affiliations:** ^1^AJA University of Medical Sciences, Tehran 1411718541, Iran; ^2^Cardiovascular Research Center, Isfahan Cardiovascular Research Institute, Isfahan University of Medical Sciences, Isfahan 81465-1148, Iran; ^3^Cardiac Rehabilitation Research Center, Isfahan Cardiovascular Research Institute, Isfahan University of Medical Sciences, Isfahan 81465-1148, Iran; ^4^Cardiac Electrophysiology Research Center, Rajaie Cardiovascular Medical and Research Center, Tehran University of Medical Sciences, Tehran 19969-11151, Iran

## Abstract

The present trial aimed to investigate the effects of pioglitazone on the serum level of asymmetric dimethylarginine (ADMA), a marker of endothelial function, and some indices of inflammation and glucose and lipid metabolism in nondiabetic metabolic syndrome patients. 104 eligible participants (57% female; age between 20 and 70) were enrolled in a double-blind placebo-controlled trial and were randomized to receive either pioglitazone (uptitrated to 30 mg/day) or matching placebo for 24 weeks. Participants were clinically examined and a blood sample was obtained at baseline and at the end of the trial. Pioglitazone significantly improved C-reactive protein level irrespective of changes in insulin sensitivity. Compared with the placebo group, alanine and aspartate transaminases were decreased and high-density lipoprotein cholesterol was increased after treatment with pioglitazone. A considerably greater weight gain was also recorded in the intervention group. We failed to observe any significant changes in serum ADMA in either group and between groups with and without adjustment for age, sex, and components of the metabolic syndrome. In a nutshell, pioglitazone seems to have positive effects on lipid profile, liver transaminases, and systemic inflammation. However, its previously demonstrated endothelial function-improving properties do not seem to be mediated by ADMA.

## 1. Introduction

Metabolic syndrome (MetS) is widely accepted as a concept which encompasses a cluster of cardiovascular risk factors. Although some authors have claimed against the additional value of the syndrome over its component parts in identifying cardiometabolic risk [1], it has been demonstrated that affected individuals are at two-to-three-fold higher risk of developing coronary heart disease and a fivefold increased risk of type-2 diabetes mellitus (T2DM) [2, 3]. Insulin resistance (IR), a key contributing mechanism to the development of the syndrome, is turned out to be associated with endothelial dysfunction (ED) [4]. In turn, ED is correlated with all risk factors of atherosclerosis [5].

ED is generally defined as the impairment of endothelium-dependent vasodilatation secondary to reduced bioavailability of nitric oxide (NO) [6]. NO production is catalyzed by the nitric oxide synthase (NOS) family of enzymes [7]. It has been shown that insulin induces endothelium-dependent vasodilatation via activating endothelial NOS [8]. Decreased bioavailability of NO mediated by endogenous inhibitors of NOS would diminish delivery of insulin and glucose to metabolically active tissues and, consequently, further aggravate the underlying IR in form of a vicious cycle [9].

Pioglitazone, a member of thiazolidinedione (TZD) family of drugs, is potent synthetic ligand for peroxisome proliferator-activated receptor gamma (PPAR-*γ*). The vascular and metabolic effects of TZDs on nondiabetic MetS patients have been the subject of a number of investigations during the recent years [10–14]. Up-to-date, molecular mediators of the ameliorating effect of pioglitazone on endothelium-dependent vasodilatation assessed by arterial flow-mediated vasodilation [10, 13–15] and venous occlusion plethysmography [16] have not thoroughly been studied. However, asymmetric dimethylarginine (ADMA), the principal endogenous NOS inhibitor, has been proposed as a possible option. Previous investigations have shown elevated levels of ADMA in non-diabetic insulin resistant subjects [17] and MetS patients [18]. This factor contradicts the vascular effects of insulin and contributes to vascular insulin resistance [19] and ED [20]. Furthermore, there is strong evidence showing that ADMA is independently correlated with total and vascular mortality [21, 22]. More significantly, it is propounded not only as a marker of disease but also as a player, and a target of pharmacologic therapy [23]. Limited available animal [24, 25] and clinical [14, 15, 17, 26] studies which evaluated the effects of TZDs on ADMA levels have produced contradictory outcomes.

This paper is presenting a part of the EPICAMP: effects of pioglitazone on cardiac structure, markers of endothelial function and psychological status study, a trial aimed to investigate the efficacy of pioglitazone monotherapy in improving a number of cardiovascular and psychiatric indices in a group of non-diabetic MetS patients. This part was designed to assess the influence of pioglitazone on the serum level of ADMA, systemic inflammation, and some indices of glucose and lipid metabolism.

## 2. Materials and Methods

### 2.1. Study Design

Eligible volunteers were enrolled in a double-blind placebo-controlled trial and were equally randomized to receive either pioglitazone (uptitrated to 30 mg/day) or matching placebo for 24 weeks. A random number list generated by random-list generator software was used in allocation of the participants. In order to observe double-blindness, the personnel who randomized patients, had no role in the data collection. For more confidentiality, we used patients' codes at all follow-up phases. Pioglitazone and identical placebo tablets were provided by Osvah Pharmaceutical Company (Tehran, Iran).

The study protocol was reviewed and approved by the Isfahan Cardiovascular Research Institute ethics committee, affiliated to Isfahan University of Medical Sciences as well as the ethics committee of AJA University of Medical Sciences. A written informed consent was also obtained from all patients after providing a brief explanation of the study method and goals. The present study was registered in the Australian New Zealand clinical trials registry (http://www.anzctr.org.au/, identifier: ACTRN12611000351910) and Iranian registry of clinical trials (http://www.irct.ir/, identifier: IRCT201101023733N2). None of the volunteers were paid for participation in the study.

### 2.2. Study Population

Study population was randomly selected among MetS patients who participated in the first phase of Isfahan Healthy Heart Program (IHHP). Details of IHHP are described elsewhere [27]. Recruitment took place in the Isfahan Cardiovascular Research Institute from March to May of 2011. Selected patients underwent screening laboratory tests as well as physical examination and structured interview by a physician. Being nondiabetic and having metabolic syndrome were our main eligibility criteria. MetS was defined following the harmonized criteria using the indices of obesity for Middle Easterns [28]. The diagnosis of T2DM was made based on the criteria provided by the American Diabetes Association [29].

Major exclusion criteria were as follows: New York Heart Association functional class 3 or 4, any history or evidences of ischemic heart disease, having polycystic ovary syndrome, impaired liver function (ALT > 2.5 times of upper limit of normal), renal dysfunction with serum creatinine >1.5 mg/dL, any debilitating medical condition, participation in a weight reduction program, being under treatment with metformin or a TZD within the preceding three months, having any of the pioglitazone contraindications, hormone replacement therapy, alcohol or drug abuse, pregnancy and lactation.

### 2.3. Efficacy and Safety Assessments

At first visit after randomization, anthropometric variables were measured according to standard methods by a trained nurse by means of a calibrated scale and an anthropometer. Waist circumference was measured in the standing position, midway between the lowest rib and iliac crest with a flexible anthropometric tape. Blood pressure was measured in the right arm at sitting position after a 10-minute rest using a standard mercury sphygmomanometer. The body mass index (BMI) was calculated using the formula: weight (kg)/height^2^ (m^2^). A structured questionnaire containing demographic data was also filled for all participants. Clinical interview and examination were done by a single trained physician at baseline and endpoint. All patients were encouraged to follow American Heart Association step 1 diet and to exercise regularly throughout the study. A blood sample was also obtained from each patient after at least 12 hours of fasting.

Patients were followed by a telephone-based interview at weeks 6 and 18 after starting of intervention performed by a physician. These observations were aimed to assess patients' adherence to medication as well as to screen for side effects. In the middle of the intervention period (week 12), all patients were visited in order to perform a general physical examination and assessment of liver transaminases. At study end, similar clinical, anthropometric, and laboratory variables to the baseline were measured. All telephone-based and face-to-face interviews were done using a preprepared check list which was designed to find any plausible side effect of pioglitazone, specially the congestive heart failure, with high sensitivity. If the occurred side effects were not indications for withdrawal, the administered drug was downtitrated to a minimum dose of 15 mg/day. Our interviewer was continually making sure that all women of childbearing potential were using an approved method of birth control throughout the study.

### 2.4. Laboratory Assays

Blood specimens were obtained at baseline and endpoint after at least 12 hours of overnight fasting. The venous blood samples were placed into tubes containing ethylene diamine tetraacetic acid and were centrifuged to separate the serum which was used to analyze the biochemical factors. A whole blood sample was also prepared. Complete blood count (CBC) with differential was done by Sysmex KX-21N (Japan) counter. Serum total, low-density and high-density lipoprotein cholesterol (LDL-C and HDL-C), triglycerides, high-sensitivity C-reactive protein (hs-CRP), creatinine, alanine transaminase (ALT) and Aspartate Transaminase (AST) levels were measured by Hitachi 902 autoanalyzer (Japan) using Pars Azmoon (Iran) analytical kits. Serum fasting glucose measurement was done using the same machine and Biosystem (France) kits. Serum insulin levels were assayed by enzyme-linked immunosorbent assay (ELISA) method using Monobind kits (CA, USA). Insulin sensitivity in the fasting state was assessed with homeostasis model assessment (HOMA) and calculated using the formula: fasting plasma glucose (milligrams per deciliter) × fasting serum insulin (microunits per milliliter)/405, as originally described by Matthews et al. [30]. A commercially available ELISA kit (DLD Diagnostika GmbH, Hamburg, Germany) was used to measure the serum levels of ADMA. Serum samples for ADMA assessment were stored at −70°C until assay. 

All the measurements were carried out in Isfahan Cardiovascular Research Institute laboratory which is qualitatively controlled by the National Reference Lab of Iran (Tehran, Iran) and INSTAND e.V. Laboratory (Düsseldorf-Germany).

### 2.5. Statistical Analysis

Baseline demographic data are presented as mean ± SD or number (percent) where appropriate. All data were assessed for a normal distribution before analyses. Skewed variables were normalized based on the Box-Cox transformation technique. As some participants were omitted from both groups, we conducted the analyses based on the intention-to-treat principles. We used mixed models for the analysis of the effects of time and group on the changes of our primary outcome variable (serum ADMA) using the STATA software (Stata/IC 9.2, StataCorp LP, TX, USA). Models were adjusted for age, sex, and all components of the MetS. Missing data were replaced by multi-imputation method using the R software (v2.9.0). Differences between baseline and posttreatment values of other variables and ADMA were analyzed using the paired Student's *t*-test. Comparisons between groups were made using the independent *t*-test for continuous and Chi-square test for discrete variables. Correlation between changes of various parameters and ADMA and CRP was studied using the Pearson correlation test. The Fisher exact test was used to evaluate the significance of difference in rate of side effects between intervention and placebo groups. The Statistical Package for Social Sciences software version 15.0 (SPSS Inc., Chicago, IL, USA) was used. A 2-tailed *P* value of ≤0.05 was considered statistically significant in all of the analyses.

## 3. Results

**Figure 1:**
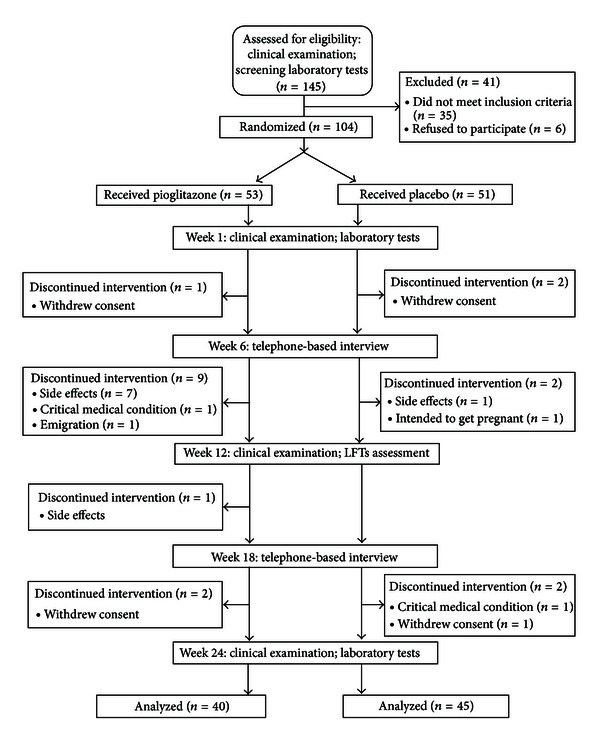
Study flow diagram.

From 145 patients screened, 104 (57% female; age between 20 and 70) were eligible and enrolled to the study. After randomized allocation, 53 patients were assigned to receive pioglitazone and 51 patients to receive placebo. Study flow diagram, which is presented in Figure 1, shows how the participants were selected and why were excluded. At the end of study, 11 patients of pioglitazone group were no longer qualified as having metabolic syndrome, compared with 7 participants receiving placebo.

### 3.1. Baseline Characteristics

Baseline characteristics of two study groups are summarized in Table 1. As shown, none of the variables were significantly different among groups before starting the intervention. Furthermore, the two groups showed similar concomitant use of various classes of antihypertensive, lipid-lowering, and weight-lowering agents at baseline and during the intervention period (data are not shown).

### 3.2. Changes in Anthropometric Variables and Blood Pressure

After 24 weeks of receiving pioglitazone, our patients had experienced a significantly higher increase of BMI versus placebo-receiving patients (*P* = 0.015). Pioglitazone and placebo-assigned groups have shown a similar significant decrease in waist circumference (−0.73% versus −1.86% resp.) and waist-to-hip ratio (−4.30% versus −4.35%, resp.), but the between-group differences never reached a significant level (Table 2).

As shown in Table 2, patients of the pioglitazone and placebo groups experienced a considerable but comparable drop in systolic (*P* < 0.001) and diastolic (*P* = 0.002 and *P* = 0.005, resp.) blood pressures.

### 3.3. Changes in Lipid Profile

After 24 weeks of intervention, the most considerable change was observed in the plasma level of HDL-C. In contrast with a nonsignificant decrease in placebo-receiving patients, HDL-C was increased significantly in intervention group (+6.81%; *P* = 0.03) and changed differently among the two groups (*P* = 0.048). Otherwise, no considerable changes were observed regarding the plasma levels of total cholesterol as well as LDL-C in either of our study groups. Similarly, experienced decreases of triglycerides in both groups compared with the baseline levels never reached our supposed level of significance (−15.85% versus −7.10%; *P* = 0.414) (Table 2).

### 3.4. Changes in Insulin Resistance

Although fasting glucose remained unchanged in both study groups, fasting serum insulin was decreased significantly in pioglitazone group (−34.01%; *P* = 0.003) and nonsignificantly in placebo group (−10.26%; *P* = 0.196), which did not reach a pronounced between-group difference (*P* = 0.137) (Table 2).

Based on our findings, pioglitazone induced a considerable decline in the degree of IR as estimated by HOMA-IR index in our patients. We have recorded a remarkable decrease of 38.96% compared with the baseline values in HOMA-IR index at the endpoint in pioglitazone-receiving patients (*P* = 0.003). Nevertheless, between-group differences of changes were not significant (Table 2).

### 3.5. Changes in Markers of Inflammation and Endothelial Function

In our study, pioglitazone-receiving patients experienced a considerable decrease of 33.44% in the serum level of CRP (*P* = 0.030), while those who received placebo have shown a modest increase of 4.87% which was significantly different (*P* = 0.040) (Table 2). Changes of CRP level correlated with the changes of neither HOMA-IR index nor fasting insulin (*r* = 0.250, *P* = 0.130 and *r* = 0.205, *P* = 0.218, resp.), indicating an independent effect. Mean peripheral WBC count was decreased in pioglitazone group (*P* = 0.038) but not differently from the placebo-assigned patients.

In our study, pioglitazone has failed to change the serum level of ADMA significantly in either of two study groups, as determined by bivariate analyses (Table 2). Similarly, none of the crude (Time: *Z* = 0.80, *P* = 0.42; Group: *Z* = −0.82, *P* = 0.41) and adjusted models for age, sex, and components of MetS (Time: *Z* = 0.78, *P* = 0.44; Group: *Z* = −0.76, *P* = 0.45) was successful in finding a significant effect of either time or group on serum levels of ADMA.

### 3.6. Safety and Tolerability

**Figure 2:**
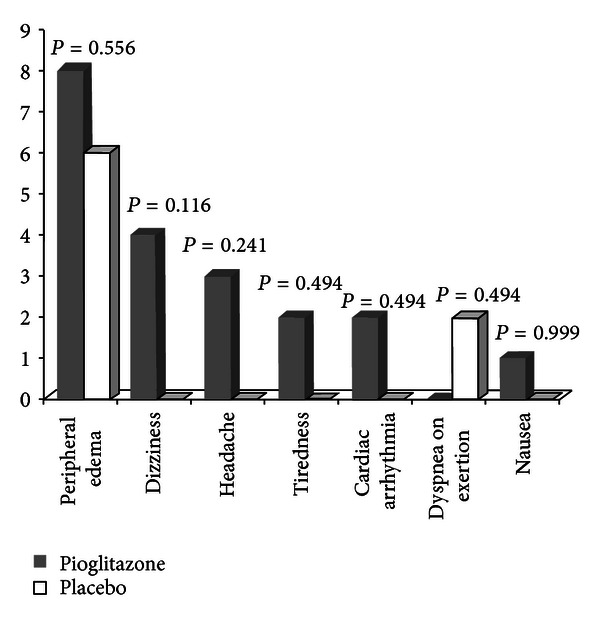
Number of participants with new-onset or aggravated signs and symptoms recorded during the follow-up period.

Experienced new-onset or aggravated signs and symptoms in pioglitazone and placebo groups are shown in Figure 2. None of the reported complications were statistically different between two groups. 

As shown in Figure 1, eight patients from the pioglitazone group and one patient from the placebo group were excluded from the study because of the side effects. One of the pioglitazone-receiving participants was shown a rise of more than 2.5 times of the upper limit of normal in ALT after 3 months and was excluded from the study. On the average, serum ALT levels were significantly decreased in pioglitazone group (*P* = 0.015) and increased in placebo group (*P* = 0.026) (Table 2). In contrast, AST levels only decreased in the pioglitazone-assigned patients (*P* ≤ 0.001). Of note, the recoded values of liver transaminases in the middle of the follow-up period were only for screening purposes and are not reported.

## 4. Discussion

The present study was a randomized placebo-controlled trial designed to assess the efficacy of pioglitazone in improving a number of vasoreactivity, inflammatory, and metabolic biomarkers.

We assessed the changes in serum levels of ADMA after 24 weeks of pioglitazone administration. Contrary to our primary hypothesis, we could not find any significant alteration in this measure. Supporting data has been obtained through a number of animal [25], in vitro, and clinical studies. Albsmeier showed that neither agonists nor antagonists of PPAR-*γ* had significant effect on cellular ADMA liberation [23]. Similarly, in a 6-month active-control trial, rosiglitazone plus metformin failed to decrease ADMA levels in diabetic patients [15]. Here a great deal of controversy emerges when the following refuting pieces of evidence are taken into consideration. TZDs were effective in reducing urine and plasma levels of ADMA in an animal study [24] and two small uncontrolled clinical trials [17, 26]. But the most reliable contradictory evidence of our results to date was derived from an 8-week rosiglitazone trial performed on 70 nondiabetic MetS patients [14]. However, the results of the present study further fueled the existing controversy rather than helping to resolve it.

Presently, there is a little doubt about the contribution of chronic subclinical inflammation in the pathophysiology of IR and MetS [31]. Moreover, it has been indicated that elevated level of CRP, as one of the most sensitive measures of inflammation, has a predictive value for the development of T2DM as well as the risk of future cardiovascular events [32]. Agonists of PPAR-*γ* have shown a wide range of anti-inflammatory properties in different clinical settings irrespective of changes in insulin sensitivity [33]. The independent CRP-lowering effect of pioglitazone shown in this study is in agreement with the findings of an absolute majority of previous investigations in diabetic and nondiabetic individuals [32]. The hypothesis that this property implicates in the reducing effect of pioglitazone on cardiac and noncardiac mortality rate [34] is waiting to be tested by future studies.

Influences of TZDs on lipid metabolism in diabetic patients have widely been investigated. Results of a meta-analysis of 23 randomized clinical trials have shown an improving effect of pioglitazone on serum triglycerides and HDL-C [35]. Nonetheless, handful number of available trials in nondiabetic insulin-resistant patients has not produced consistent results. Our study, the largest so far in terms of the sample size, in line with three other trials [10, 12, 36] has found pioglitazone to have a significant increasing effect on serum HDL-C but not on triglycerides. In contrast, others [37–39] have failed to observe any considerable change in lipid profile of nondiabetic participants after 12–16 weeks of pioglitazone administration. While these results await future confirmation, the null effect of pioglitazone on total cholesterol and LDL-C in our study seems undisputed as no contradictory reports have been found.

We have observed a significantly higher increase in BMI in pioglitazone-treated patients. The recorded mean increase of 2.73 kg in our patients was within the previously reported range of weight gain induced by TZD compounds in diabetic patients (from 2.0 to 4.3 kg) [40]. However, results in nondiabetic individuals have been mixed as some smaller-sized trials failed to document a significant effect of pioglitazone on body weight [12, 36, 38]. Fluid retention, differentiation of adipocytes, increased body fat, and increased appetite are among mechanisms which were supposed to be responsible for the weight gain in TZD users [41]. 

Surprisingly, the indices of central adiposity and blood pressure were diminished similarly in our both study groups. The authors suppose that this finding could be the consequence of sustained blood pressure management and lifestyle modification advice given to the participants throughout the follow-up period, and it could not be attributed to the interventions.

As expected, pioglitazone has caused a significant improvement in surrogate indicators of insulin sensitivity irrelevant of its effects on fasting glucose level. But the HOMA-IR index and fasting serum insulin were also decreased in our placebo group to a nonsignificant but enough degree to prevent the between-group difference of changes from reaching a significant level. We suppose that this finding could be interpreted by the observed decline in our patients' waist circumference. Considering the fact that waist circumference is linearly related to IR [42] and reduction of this measure of visceral adiposity could lead to an improvement in insulin sensitivity [43]. These outcomes highlight the efficacy of lifestyle modification advice in improving IR in MetS patients.

The present study has evidenced the beneficial effects of pioglitazone on both ALT and AST levels in non-diabetic MetS patients. This effect is considerable as ALT has displayed an inverse relationship with cardiovascular and non-cardiovascular events in population-based studies [44]. Clarification of the mechanisms needs more detailed studies. Overall, our trial was not powered to reliably investigate the medication's safety. Accordingly and with regard to the paucity of papers reporting the side effect profile of TZDs in non-diabetic insulin-resistant patients, making definite statements is impossible at the present stage. 

In spite of benefiting from being randomized, controlled, and having a proper duration of intervention, the validity of our results is affected by some limitations. First is the fact that we used a static index to estimate IR rather than a better-validated direct measure such as hyperinsulinemic euglycemic glucose clamp. Additionally, it seems that repeated measurement of the metabolic biomarkers would provide the possibility of extracting pathophysiologic clues from the trend of induced changes, which was not performed in this study. Finally, the design of this study would have been more favorable if direct measures of endothelial function had been used concomitantly with the biomarkers.

Considering our findings, pioglitazone had positive effects on insulin sensitivity, lipid profile, liver transaminases, and systemic inflammation, despite inducing an increase in BMI and failure in making a significant change in serum level of ADMA. In conclusion, this study has added to the priory obtained evidence that pioglitazone could be considered in non-diabetic MetS patients with preserved cardiac function who failed to follow the life style modification recommendations.

## Figures and Tables

**Table 1:** Baseline characteristics of participants^a,b^.

	Pioglitazone (*n* = 53)	Placebo (*n* = 51)	*P* value
Male sex	19 (35.8%)	25 (49.0%)	0.306
Age (years)	49.8 ± 10.05	50.9 ± 10.11	0.599
Education			0.442
Undergraduate	46 (86.8%)	44 (83.3%)	
Graduate	7 (13.2%)	7 (13.7%)	
Current smoker^c^	10 (18.9%)	4 (7.8%)	0.108
Body mass index (kg/m^2^)	30.26 ± 3.23	30.06 ± 4.20	0.809
Waist circumference (cm)	102.72 ± 9.26	101.74 ± 10.18	0.652
Waist-to-hip ratio	0.97 ± 0.07	0.96 ± 0.07	0.656
Blood pressure (mmHg)			
Systolic	129.22 ± 14.87	134.23 ± 21.14	0.262
Diastolic	82.50 ± 8.33	85.64 ± 10.21	0.166
Fasting glucose (mg/dL)	94.10 ± 11.17	95.12 ± 11.20	0.106
Serum lipids (mg/dL)			
Total cholesterol	204.65 ± 31.10	215.80 ± 40.41	0.184
HDL-C	42.00 ± 10.52	43.05 ± 8.68	0.631
LDL-C	110.18 ± 19.60	118.15 ± 29.54	0.185
Triglycerides	226.54 ± 145.53	202.97 ± 108.41	0.429
Liver transaminases (U/L)			
ALT	29.97 ± 12.99	26.53 ± 12.09	0.232
AST	26.92 ± 6.28	25.25 ± 6.16	0.247
White blood cells (10^3^/mL)	6.25 ± 1.40	6.58 ± 1.36	0.396
hs-CRP (mg/L)	3.14 ± 2.03	2.26 ± 1.25	0.070
Fasting insulin (*μ*U/mL)	13.24 ± 5.74	13.43 ± 7.96	0.913
HOMA-IR index	3.21 ± 1.38	3.18 ± 2.05	0.937
ADMA (*μ*m/L)	0.64 ± 0.36	0.70 ± 0.40	0.652

^a^Data are expressed as mean ± SD for continuous variables and number (percentage) of participants for categorical variables.

^b^Abbreviations are defined in the text.

^c^Was defined as one who regularly smoked at least one cigarette per day.

**Table 2:** Analysis of changes in assessed variables in pioglitazone and placebo groups^a^.

Variable (mean ± SD)	Pioglitazone			Placebo			Between-group comparisons	
	Baseline (*n* = 53)	Week 24 (*n* = 40)	*P* value	Baseline (*n* = 51)	Week 24 (*n* = 45)	*P* value	Corrected difference (95% CI)	*P* value
Body mass index (kg/m^2^)	30.26 ± 3.23	31.29 ± 3.84	0.451	30.06 ± 4.20	30.36 ± 4.16	0.103	+.73 (0.1 to 1.3)	**0.015**
Waist-circumference (cm)	102.72 ± 9.26	101.97 ± 10.17	**<0.001**	101.74 ± 10.18	99.58 ± 10.25	**0.003**	+1.42 (−0.9 to 3.8)	0.228
Waist-to-hip ratio	0.97 ± 0.07	0.93 ± 0.08	**<0.001**	0.96 ± 0.07	0.92 ± 0.06	**<0.001**	+.004 (−0.02 to 0.03)	0.712
Blood pressure (mmHg)								
Systolic	129.22 ± 14.87	117.50 ± 10.40	**<0.001**	134.23 ± 21.14	124.10 ± 14.82	**<0.001**	−1.59 (−8.0 to 4.8)	0.623
Diastolic	82.50 ± 8.33	77.97 ± 4.55	**0.002**	85.64 ± 10.21	81.41 ± 7.52	**0.005**	−.30 (−4.3 to 3.7)	0.882
Serum lipids (mg/dL)								
Total cholesterol	204.65 ± 31.10	210.46 ± 36.09	0.267	215.80 ± 40.41	210.03 ± 39.98	0.218	+11.58 (−2.2 to 25.3)	0.097
HDL-C	42.00 ± 10.52	44.86 ± 11.15	**0.030**	43.05 ± 8.68	42.85 ± 10.32	0.826	+3.06 (0.02 to 6.1)	**0.048**
LDL-C	110.18 ± 19.60	114.85 ± 23.00	0.187	118.15 ± 29.54	121.33 ± 29.21	0.293	+1.50 (−7.6 to 10.6)	0.743
Triglycerides	226.54 ± 145.53	195.54 ± 107.88	0.053	202.97 ± 108.41	189.51 ± 75.76	0.365	−17.54 (−60.1 to 25.0)	0.414
Liver transaminases (U/L)								
ALT	29.97 ± 12.99	24.95 ± 8.89	**0.015**	26.53 ± 12.09	30.15 ± 15.30	**0.026**	−8.65 (−13.6 to −3.7)	**0.001**
AST	26.92 ± 6.28	22.44 ± 4.76	**<0.001**	25.25 ± 6.16	24.85 ± 7.83	0.711	−4.07 (−7.2 to −1.0)	**0.010**
Fasting glucose (mg/dL)	98.57 ± 13.10	97.78 ± 19.05	0.782	94.10 ± 11.17	95.12 ± 11.20	0.478	−1.81 (−7.9 to 4.3)	0.555
Fasting insulin (*μ*U/mL)	13.24 ± 5.74	9.88 ± 4.02	**0.003**	13.43 ± 7.96	12.18 ± 6.08	0.196	−2.11 (−4.9 to 0.7)	0.137
HOMA-IR index	3.21 ± 1.38	2.31 ± 1.02	**0.003**	3.18 ± 2.05	2.94 ± 1.67	0.334	−0.67 (−1.4 to 0.1)	0.077
White blood cells (10^3^/mL)	6.25 ± 1.40	5.81 ± 1.09	**0.038**	6.58 ± 1.36	6.31 ± 1.15	0.135	−0.17 (−0.7 to 0.4)	0.530
hs-CRP (mg/L)	3.14 ± 2.03	2.09 ± 1.02	**0.030**	2.26 ± 1.26	2.37 ± 1.42	0.743	−1.16 (−2.3 to −0.1)	**0.040**
ADMA (*μ*m/L)	0.64 ± 0.36	0.61 ± 0.16	0.675	0.70 ± 0.40	0.63 ± 0.13	0.316	+0.02 (−0.2 to 0.2)	0.812

^a^Abbreviations are defined in the text.
